# Supplementary material for: Acute Kidney Injury in Children with Polyuria: A Systematic Review
Source: J Clin Med. 2026 Jan 2;15(1):351. doi: 10.3390/jcm15010351 (PMC12786687; doi:10.3390/jcm15010351)
Supplement: Supplementary file 1 [file jcm-15-00351-s001.zip › Supplementary File S2.pdf]

Supplementary File 2: List of studies not included and exclusion reasons.

Authors	Title	Year	DOI	Exclusion Criteria
Chen H. *et al.*	Short-term intra-individual variation of urinary biomarkers in dogs with stable chronic kidney disease	2023	10.1111/jvim.16619	No keyword
Goggs R. *et al.*	Serial analysis of blood biomarker concentrations in dogs with pneumonia	2022	10.1111/jvim.16374	No keyword
Rodrigues P. *et al.*	Clinical phenotyping of children with nocturnal enuresis: A key classification to improve the approach	2024	10.1016/j.jpurol.2024.01.019	Review
Golightly L.K. *et al.*	Renal pharmacotherapy: Dosage adjustment of medications eliminated by the kidneys	2021	10.1007/978-3-030-58650-8	Book
Kanda J. *et al.*	Recurrent transient severe hypocalcaemia in two siblings with type 1 Bartter syndrome	2024	10.1111/nep.14261	No keyword
St Pierre K. *et al.*	Interventions for preventing the progression of autosomal dominant polycystic kidney disease	2024	10.1002/14651858.CD010294.pub3	Review
Roy A. *et al.*	Pituitary Dysfunction Following Snakebite Envenomation: A Clinico-Radiological Assessment of 15 Cases and Review of the Literature	2022	10.4103/0028-3886.359201	Review
Hosseini S. *et al.*	Evaluating the effects of intravenous magnesium sulfate for prevention of colistin induced acute kidney injury: an open-label	2024	10.1007/s00210-024-03583-w	No keyword
Crawford B. *et al.*	Native nephrectomy in advanced pediatric kidney disease: indications	2024	10.1007/s00467-023-06117-3	Review
Meneghel A. *et al.*	Renal Involvement in Multisystem Inflammatory Syndrome in Children: Not Only Acute Kidney Injury	2023	10.3390/children10101661	No keyword
An J. *et al.*	Regulation of intestinal micro ecology between raw and salt-processed Alpinia oxyphylla on renal injury rats	2023	10.36721/PJPS.2023.36.2.REG.557-564.1	No keyword
Levy Z. *et al.*	Absolute Neurocritical Care Review	2023	10.1007/978-3-031-24830-6	Book
Rahimi R.A. *et al.*	A comprehensive review of the use of Sodium Chloride (NaCl) in the development of the COVID-19 vaccine and medical applications	2023	10.31893/multirev.2023028	Review
Ushijima-Fuchino K. *et al.*	Urinary stone in a 12-year-old adolescent with new-onset type 1 diabetes and diabetic ketoacidosis	2022	10.1297/cpe.2021-0069	No keyword
Janssens P. *et al.*	The emerging role of the apelinergic system in kidney physiology and disease	2022	10.1093/ndt/gfab070	Review
Kuok C.I. *et al.*	Unusual course in minimal change nephropathy: Exaggerated polyuria after anuric acute kidney injury	2022	10.1111/jpc.15706	No keyword
Lebensburger J. *et al.*	The Kidney in Sickle Cell Disease	2023	10.1007/978-3-031-11665-0_31	Book
Mboni-Johnston I.M. *et al.*	Sensitivity of Human Induced Pluripotent Stem Cells and Thereof Differentiated Kidney Proximal Tubular Cells towards Selected Nephrotoxins	2024	10.3390/ijms25010081	No keyword
Kojc T.S. *et al.*	Novel Biomarkers of Heart Failure in Pediatrics	2022	10.3390/children9050740	Review
Hammi Y. *et al.*	Genotype-Phenotype correlation of distal renal tubular acidosis in Tunisia	2023		No keyword
Abdelrahaman D. *et al.*	Nootkatone Counteracts Melamine-Mediated Nephrotoxicity via Modulation of Intermediate Filament Proteins	2024	10.2147/DDDT.S466286	No keyword
Yang C. *et al.*	Jiawei Shengjiangsan's Effect on Renal Injury in Diabetic Nephropathy Mice is Investigated via the PI3K/Akt/NF-κB Signaling Pathway	2024	10.2147/DMSO.S456205	No keyword
Taylor B. *et al.*	Renal safety of critical care sedation with sevoflurane: a systematic review and meta-analysis	2023	10.1007/s00540-023-03227-y	Review
Peters N.J. *et al.*	Perioperative fluid and electrolyte management in surgical neonates	2023	10.47338/jns.v13.1225	Review
Junaid K. *et al.*	Prevalence, Clinical Characteristics, and Clinical Outcomes of New-onset Diabetes Mellitus among COVID-19 Patients in Developing and Developed Countries: A Systematic Review	2023	10.29271/jcpsp.2023.06.691	Review
Igarashi T. *et al.*	Pediatric Fanconi Syndrome	2022	10.1007/978-3-030-52719-8_38	Book
Das L. *et al.*	Unusual and lesser-known rare causes of adult growth hormone deficiency	2023	10.1016/j.beem.2023.101820	Review
Yaman A. *et al.*	Severe Rhabdomyolysis and Acute Renal Failure Treated by Continuous Venovenous Hemodiafiltration in a Child with Diabetic Ketoacidosis	2022	10.5005/jp-journals-10071-24093	No keyword
Sartorius V. *et al.*	Post-obstructive diuresis after posterior urethral valve treatment in neonates: a retrospective cohort study	2024	10.1007/s00467-023-06100-y	No keyword
Afzal S. *et al.*	Hantavirus: an overview and advancements in therapeutic approaches for infection	2023	10.3389/fmicb.2023.1233433	Review
Skrajnowska D. *et al.*	The Effects of Diet, Dietary Supplements, Drugs and Exercise on Physical, Diagnostic Values of Urine Characteristics	2024	10.3390/nu16183141	Review
Giglio R.V. *et al.*	Novel molecular markers of cardiovascular disease risk in type 2 diabetes mellitus	2021	10.1016/j.bbadis.2021.166148	Review
Gorvin C.M. *et al.*	Genetic causes of neonatal and infantile hypercalcaemia	2022	10.1007/s00467-021-05082-z	Review
Nørregaard R. *et al.*	Obstructive nephropathy and molecular pathophysiology of renal interstitial fibrosis	2023	10.1152/PHYSREV.00027.2022	Review
Hart D.A *et al.*	Lithium Ions as Modulators of Complex Biological Processes: The Conundrum of Multiple Targets	2024	10.3390/biom14080905	Review
Leuba C. *et al.*	Post-obstructive diuresis, by the internal physician	2023	10.1016/j.revmed.2023.01.011	Review
Reddi A.S. *et al.*	Absolute Nephrology Review: An Essential Q & A Study Guide	2022	10.1007/978-3-030-85958-9	Book
Rozenfeld P. *et al.*	The role of tubular cells in the pathogenesis of Fabry nephropathy	2024	10.3389/fcvm.2024.1386042	Review
De Leo E. *et al.*	Genistein improves renal disease in a mouse model of nephropathic cystinosis: a comparison study with cysteamine	2023	10.1093/hmg/ddac266	No keyword
Sun Y. *et al.*	The Utility of Exosomes in Diagnosis and Therapy of Diabetes Mellitus and Associated Complications	2021	10.3389/fendo.2021.756581	Review
Sykes J.E. *et al.*	Updated ACVIM consensus statement on leptospirosis in dogs	2023	10.1111/jvim.16903	No keyword

Papaetis G.S. *et al.*	Empagliflozin and the diabetic kidney: Pathophysiological concepts and future challenges	2021	10.2174/1871530321999201214233421	Review
Hammi Y. *et al.*	Phenotype Spectrum in Tunisian Population with NPHP1 Deletion	2023	10.4103/ijn.ijn_248_22	No keyword
Medeiros M. *et al.*	Proximal renal tubular acidosis (Type II)	2022	10.1007/978-3-030-91940-5_8	Book
Ericksen P.N. *et al.*	Standardization of coding definitions for sickle cell disease complications: A systematic literature review	2024	10.1002/pds.5769	Review
Miguel P.J. *et al.*	Paracetamol Induced Acute Interstitial Nephritis: A Pediatric Case Report	2024	10.20344/amp.20563	Letter
Filler G. *et al.*	Animal, Human, and 23Na MRI Imaging Evidence for the Negative Impact of High Dietary Salt in Children	2021	10.1007/s40124-021-00249-6	Review
Alexander S.M. *et al.*	A 5-year-old with new-onset diabetes presenting with ketoacidosis	2022	10.1177/2050313X221130582	No keyword
Singh A. *et al.*	A novel study on the quality of life index in canine chronic kidney disease treated with incremental intermittent hemodialysis	2024	10.14202/vetworld.2024.1702-1714	No keyword
Conte M.L. *et al.*	Moyamoya disease presenting with tubular dysfunction in a child: pitfalls in diagnosing an atypical hyponatremic-hypertensive syndrome	2023	10.1186/s12887-023-03926-1	No keyword
Greenbaum L.A. *et al.*	Maintenance and Replacement Therapy	2024	10.1016/B978-0-323-88305-4.00074-2	Book
Ali S. *et al.*	A novel case of lupus nephritis and mixed connective tissue disorder in a COVID-19 patient	2022	10.1016/j.amsu.2022.103653	No keyword
Liu X. *et al.*	Acute pancreatitis associated with diabetic ketoacidosis in a child with COVID-19 infection	2023	10.1186/s12879-023-08371-0	No keyword
Dutta A. *et al.*	Deciphering the role of aquaporins in metabolic diseases: A mini review	2022	10.1016/j.amjms.2021.10.029	Review
De Wall L.L. *et al.*	Outcome of a thorough screening of lower urinary tract function in all pediatric kidney recipients	2021	10.1111/petr.13929	No keyword
Huang V.W. *et al.*	Kidney disease progression in pediatric and adult posterior urethral valves (PUV) patients	2024	10.1007/s00467-023-06128-0	No keyword
Bezruk V. *et al.*	Clinical diagnostic algorithms of action in the practice of a doctor- Family medicine diseases of the urinary system in children	2024	10.24061/2413-4260.XIV.1.51.2024.23	No English
Damba J.J. *et al.*	Psychotropic Drugs and Adverse Kidney Effects: A Systematic Review of the Past Decade of Research	2022	10.1007/s40263-022-00952-y	Review
Layne K. *et al.*	100 Diagnostic Dilemmas in Clinical Medicine: Second Edition	2023	10.1201/9781003344117	Book
Qiu Z. *et al.*	Aquaporins in Urinary System	2023	10.1007/978-981-19-7415-1_11	Book
Kostopoulou E. *et al.*	Diabetic Ketoacidosis in Children and Adolescents; Diagnostic and Therapeutic Pitfalls	2023	10.3390/diagnostics13152602	Review
Angelousi A. *et al.*	New developments and concepts in the diagnosis and management of diabetes insipidus (AVP-deficiency and resistance)	2023	10.1111/jne.13233	Review
Hoang S. *et al.*	A case of ifosfamide-induced acute kidney injury	2024	10.1007/s13730-023-00829-z	No keyword
Fischer Sigel L.K. *et al.*	Progression of renal damage and tubular regeneration in pregnant and non-pregnant adult female rats inoculated with a sublethal dose of Shiga toxin 2	2024	10.1016/j.micpath.2023.106482	No keyword
Ruttkowski L. *et al.*	Case report: Febrile infection-related epilepsy syndrome in a 14-year-old girl with multiple organ failure and lethal outcome	2024	10.3389/fnins.2024.1255841	No keyword
Branco B.C. *et al.*	Novel Biomarkers for Posterior Urethral Valve	2023	10.2174/0929867329666220803120302	Review
Farooq U. *et al.*	Is acid suppression associated with the increased length of stay in diabetic ketoacidosis patients? A nationwide analysis	2022	10.1111/joim.13466	No keyword
Brown T. *et al.*	Determination of the reference interval for urine kidney injury molecule-1 in 50 healthy cats	2024	10.1177/1098612X241238923	No keyword
Zhang A. *et al.*	Clinical practice guideline for early screening of pediatric chronic kidney disease in China	2022	10.3760/cma.j.cn112140-20220714-00647	No English
Mistry A.M. *et al.*	Which Intravenous Isotonic Fluid Offers Better Outcomes for Patients with a Brain Injury?	2024	10.1007/s12028-024-02139-3	No keyword
Marzuillo P. *et al.*	Renal involvement in children with type 2 diabetes mellitus onset: A pilot study	2021	10.3390/children8080627	No keyword
Hollywood J.A. *et al.*	Cystinosin-deficient rats recapitulate the phenotype of nephropathic cystinosis	2022	10.1152/ajprenal.00277.2021	No keyword
Thakare S.B. *et al.*	Acquired disorders of phosphaturia: Beyond tumor-induced osteomalacia	2024	10.1016/j.beem.2023.101839	Review
Zaheer J. *et al.*	99mTc-DMSA and 99mTc-DTPA identified renal dysfunction due to microplastic polyethylene in murine model	2024	10.1016/j.chemosphere.2024.143108	No keyword
Elinder C.G. *et al.*	Renal effects of exposure to metals	2021	10.1016/B978-0-12-823292-7.00013-9	Book
Choo D. *et al.*	Ultrasonographic quantitative evaluation of acute and chronic renal disease using the renal cortical thickness to aorta ratio in dogs	2023	10.1111/vru.13154	No keyword
Rivetti G. *et al.*	Editorial on the Special Issue “Advances in Pediatric Acute Kidney Injury”	2024	10.3390/children11020195	Editorial
Taravilla C.N. *et al.*	Deferasirox and complex proximal tubulopathy. Presentation of two clinical cases	2021	10.32641/ANDESPEDIATR.V92I4.3154	No English
Kurtz M. *et al.*	Usefulness of serum amyloid A for the diagnosis of pyelonephritis in cats: A prospective evaluation	2024	10.1111/jvim.17082	No keyword
Bayani M. *et al.*	The nosocomial infection survey among patients suffering from the Coronavirus disease-2019 hospitalized in Ayatollah Rouhani Hospital	2024	10.22088/cjim.15.3.509	No keyword
Reddi A.S. *et al.*	Fluid, Electrolyte and Acid-Base Disorders	2023	10.1007/978-3-031-25810-7	Book
Weber D.R. *et al.*	Diagnostic Approach and Treatment of the Pediatric Patient with Hypercalcemia	2022	10.1007/978-3-030-93182-7_4	Book
Concepción-Zavaleta M. *et al.*	Ifosfamide-induced nephrogenic diabetes insipidus and Fanconi syndrome in a patient with femur osteosarcoma	2024	10.22088/cjim.15.4.743	No keyword
AbdelRazek M. *et al.*	Primary versus deferred ureteroscopy for the management of obstructive anuria secondary to ureteric urolithiasis in children: a prospective randomized study	2023	10.1007/s00240-022-01389-0	No keyword
Shen I. *et al.*	A potential novel treatment for cirrhosis-related ascites: Empagliflozin is safe and tolerable in advanced chronic liver disease	2024	10.1111/bcp.16139	No keyword

Cano F. *et al.*	Normal Reference Values	2022	10.1007/978-3-030-52719-8_142	Book
Petrea C.L. *et al.*	A Study of Hydroelectrolytic and Acid–Base Disturbances in MIS-C Patients: A Perspective on Antidiuretic Hormone Secretion	2024	10.3390/cimb46100681	No keyword
Rizzo M. *et al.*	Bilateral Global Nephrocalcinosis in a Uremic Puppy	2024	10.3390/vetsci11080338	No keyword
Çelik T. *et al.*	Evaluation of Childhood Fever Management	2024	10.5578/ced.20240102	No keyword
Vijayakanthi N. *et al.*	Rhabdomyolysis due to rosuvastatin in a patient with ROHHAD syndrome	2021	10.1016/j.jacl.2021.09.002	No keyword
Najafi R. *et al.*	A challenging case of COVID-19: a COVID-19 positive adolescent presented with severe diabetic ketoacidosis	2022	10.1186/s12902-022-00979-8	No keyword
Sengupta A. *et al.*	Transient Fanconi Syndrome in a Child with Acute COVID-19 Infection	2021	10.1007/s12098-021-03890-7	Letter
Dionne J.M. *et al.*	Neonatal Hypertension	2023	10.1016/B978-0-323-69415-5.00060-6	Book
Lunati M.E. *et al.*	SGLT2-inhibitors are effective and safe in the elderly: The SOLD study	2022	10.1016/j.phrs.2022.106396	No keyword
Zand Irani A. *et al.*	Immune checkpoint inhibitor-induced diabetes mellitus with pembrolizumab	2022	10.1136/bcr-2021-245846	No keyword
Kubanek A. *et al.*	Acute Kidney Injury and Post-Artesunate Delayed Haemolysis in the Course of Plasmodium falciparum Malaria	2024	10.3390/pathogens13100851	No keyword
Huang M. *et al.*	Clinicopathological features and short outcomes of oliguric acute tubular injury	2022	10.1016/j.jcrc.2022.154076	No keyword
Kim J.E. *et al.*	Effects of Multiwall Carbon Nanotubes on Premature Kidney Aging: Biochemical and Histological Analysis	2023	10.3390/toxics11040373	No keyword
D'Alessandri-Silva C. *et al.*	Diagnosis, Treatment, and Outcomes in Children With Congenital Nephrogenic Diabetes Insipidus: A Pediatric Nephrology Research Consortium Study	2020	10.3389/fped.2019.00550	No keyword
Langston C. *et al.*	Effects of IV Fluids in Dogs and Cats With Kidney Failure	2021	10.3389/fvets.2021.659960	Review
Chapman C.L. *et al.*	Kidney physiology and pathophysiology during heat stress and the modification by exercise	2021	10.1080/23328940.2020.1826841	Review
Radi Z.A. *et al.*	Cardio-renal safety of non-steroidal anti-inflammatory drugs	2019	10.2131/jts.44.373	Review
Fountas A. *et al.*	Hypopituitarism	2018	10.1016/B978-0-12-801238-3.64277-0	Book
Magdalan J. *et al.*	Acute intranasal intoxication with mercuric chloride taken accidently instead of cocaine - A case report	2021	10.1016/j.jflm.2021.102129	No keyword
Schreuder M.F *et al.*	Life with one kidney	2018	10.1007/s00467-017-3686-4	Review
Sobieszczańska-Droździel A. *et al.*	Acute tubulointerstitial nephritis in 10-year-old boy following severe acute respiratory syndrome coronavirus 2 infection	2021	10.5114/POLP.2021.110553	No English
Chávez-Iñiguez J.S. *et al.*	Acute kidney injury caused by obstructive nephropathy	2020	10.1155/2020/8846622	Review
Tuli G. *et al.*	Clinical Approach to Sodium Homeostasis Disorders in Children with Pituitary-Suprasellar Tumors	2020	10.1159/000502609	Review
Upadhyay A. *et al.*	Ibuprofen-based advanced therapeutics: breaking the inflammatory link in cancer	2021	10.1080/03602532.2021.1903488	Review
Regev L.C. *et al.*	A riddle wrapped in an enigma: acute kidney injury in a girl with Crohn's disease: Answers	2020	10.1007/s00467-020-04538-y	Answer
Anon B. *et al.*	Ibuprofen-based advanced therapeutics: breaking the inflammatory link in cancer	2017	10.1016/j.arcped.2017.05.012	Short survey
Mansoor A.M. *et al.*	Frameworks for Internal Medicine	2018		Book
Wang A. *et al.*	Hydrochlorothiazide ameliorates polyuria caused by tolvaptan treatment of polycystic kidney disease in PCK rats	2019	10.1007/s10157-018-1669-9	No keyword
Sprangers B. *et al.*	Conventional chemotherapy	2019	10.1016/B978-0-323-54945-5.00025-4	Book
Graeme K.A. *et al.*	Nonsteroidal anti-inflammatory drugs	2017	10.1007/978-3-319-17900-1_96	Book
Henry R.K. *et al.*	Hypercalcemia due to milk-alkali syndrome and fracture-induced immobilization in an adolescent boy with hypoparathyroidism	2016	10.1159/000446316	No keyword
Shah S.K. *et al.*	Hypophosphatemia in Critically Ill Children: Risk Factors	2016	10.1007/s12098-016-2188-x	No keyword
García-Ortuño L.E. *et al.*	Integrative view of the mechanisms that induce acute kidney injury and its transition to chronic kidney disease	2018	10.24875/RIC.18002546	Review
Górriz J.L. *et al.*	Sodium-glucose cotransporter 2 inhibition: Towards an indication to treat diabetic kidney disease	2020	10.1093/NDT/GFZ237	Review
Mctaggart J.S. *et al.*	Type 1 diabetes mellitus presenting with diabetic ketoacidosis in a child with Patau syndrome (trisomy 13) and persistent fetal haemoglobin	2021	10.1136/bcr-2021-243077	No keyword
Tan Y.G. *et al.*	Hypertensive urgency in nephrogenic diabetes insipidus with concomitant Hinman syndrome	2019	10.1136/bcr-2018-229095	No keyword
Weir M.A. *et al.*	Antiinflammatory Drugs and the Kidney	2019	10.1016/B978-0-323-44942-7.00218-1	Book
Basmaji J. *et al.*	A systematic review of donor serum sodium level and its impact on transplant recipients	2020		Review
Winterberg P.D. *et al.*	Kidney transplantation in children	2019	10.1016/B978-0-323-53186-3.00037-1	Book
Rangan G. *et al.*	Current and emerging treatment options to prevent renal failure due to autosomal dominant polycystic kidney disease	2020	10.1080/21678707.2020.1804859	Review
Hutchinson K.M. *et al.*	A Review of Central Venous Pressure and Its Reliability as a Hemodynamic Monitoring Tool in Veterinary Medicine	2016	10.1053/j.tcam.2016.08.009	Review
Stokes V.J. *et al.*	Hypercalcemic Disorders in Children	2017	10.1002/jbmr.3296	Review
Misgar R.A. *et al.*	Vitamin D toxicity: A prospective study from a tertiary care centre in Kashmir Valley	2019	10.4103/ijem.ijem_116_19	No keyword
Amerio A. *et al.*	Safety and efficacy of lithium in children and adolescents: A systematic review in bipolar illness	2018	10.1016/j.eurpsy.2018.07.012	Short survey

Gupta M. *et al.*	Vasoactive Substances as Mediators of Renal Injury	2018	10.1016/B978-0-08-100601-6.00810-3	Book
Leipziger J. *et al.*	Renal autocrine and paracrine signaling: A story of self-protection	2020	10.1152/physrev.00014.2019	Review
Neugarten J. *et al.*	Nephrotoxicity of Lithium and Drugs of Abuse	2018	10.1016/B978-0-12-801238-3.02057-2	Book
Downie M.L. *et al.*	A curious case of growth failure and hypercalcemia: Answers	2018	10.1007/s00467-017-3769-2	No keyword
Ma Y. *et al.*	Comparison of diabetic nephropathy between male and female eNOS-/- db/db mice	2019	10.1152/ajprenal.00023.2019	No keyword
Uchida Y. *et al.*	Furosemide as a factor to deteriorate therapeutic efficacy of tolvaptan in patients with decompensated cirrhosis	2020	10.1111/hepr.13566	No keyword
Barrell E.A. *et al.*	Diseases of the Renal System	2019	10.1016/B978-0-323-55445-9.00034-3	Book
Mrad F.C.C. *et al.*	Bartter's syndrome: clinical findings	2021	10.1007/s12519-020-00370-4	Review
Moran P. *et al.*	Dysnatremias-what causes them and how should they be treated?	2019	10.1016/B978-0-323-64068-8.00070-5	Book
Attini R. *et al.*	Dialysis or a plant-based diet in advanced ckd in pregnancy? A case report and critical appraisal of the literature	2019	10.3390/jcm8010123	No keyword
de Oliveira Campos J.L. *et al.*	Renovascular hypertension in pediatric patients: update on diagnosis and management	2020	10.1007/s00467-021-05063-2	Review
Fiscaletti M. *et al.*	Two Cases of Mistaken Polyuria and Nephrocalcinosis in Infants with Glucose-Galactose Malabsorption: A Possible Role of 1	2017	10.1159/000454951	No keyword
Pecchiari M. *et al.*	Cardiovascular Responses During Sepsis	2021	10.1002/cphy.c190044	No keyword
Hazelhoff M.H. *et al.*	Trimetazidine Protects from Mercury-Induced Kidney Injury	2021	10.1159/000514843	No keyword
Pedraza Bermeo A.M. *et al.*	Risk factors for postobstructive diuresis in pediatric patients with ureteropelvic junction obstruction	2018	10.1016/j.jpurol.2018.01.017	No keyword
Dobrek Ł. *et al.*	The influence of oxazaphosphorine agents on kidney function in rats	2017	10.1016/j.medici.2017.05.004	No keyword
Howard S.C. *et al.*	Preventing andmanaging toxicities of high-dose methotrexate	2016	10.1634/theoncologist.2015-0164	No keyword
Thakore P. *et al.*	Central diabetes insipidus: A rare complication of IVH in a very low birth weight preterm infant	2019	10.3233/NPM-1837	No keyword
Nagler E.V. *et al.*	Interventions for chronic non-hypovolaemic hypotonic hyponatraemia	2018	10.1002/14651858.CD010965.pub2	Review
Li Z. *et al.*	Melatonin therapy protects against renal injury before and after release of bilateral ureteral obstruction in rats	2019	10.1016/j.lfs.2019.05.034	No keyword
McMahon K.R. *et al.*	Design and methods of the pan-canadian applying biomarkers to minimize long-term effects of childhood/adolescent cancer treatment (ABLE) nephrotoxicity study: A prospective observational cohort study	2017	10.1177/2054358117690338	No keyword
Liu Y. *et al.*	Shiga toxins: An update on host factors and biomedical applications	2021	10.3390/TOXINS13030222	No keyword
McKenna M. *et al.*	Clinical utility of estimation of glomerular filtration rate in dogs	2020	10.1111/jvim.15561	No keyword
Krishnamurthy A. *et al.*	Anticancer medications and sodium dysmetabolism	2020	10.17925/ee.2020.16.2.122	Review
Shakoor J. *et al.*	Clinical and morphological spectrum of congenital anomalies – A Tertiary care center experience	2020		No keyword
Payus A.O. *et al.*	Cranial diabetes insipidus in neuropsychiatrie systemic lupus erythematosus	2020	10.12996/gmj.2020.48	No keyword
Langston C. *et al.*	Managing Fluid and Electrolyte Disorders in Kidney Disease	2017	10.1016/j.cvsm.2016.09.011	Review
Prometnoi D.V. *et al.*	Fluid overload as a predictor of lethal outcome in critically-Ill children	2019	10.15360/1813-9779-2019-1-12-26	No keyword
Imaizumi T. *et al.*	Glucocorticoid treatment is associated with ICU-acquired hypernatremia: a nested case–control study	2021	10.1007/s10157-020-01967-9	No keyword
Kim Y.M. *et al.*	A case of an infant suspected as IMAGE syndrome who were finally diagnosed with MIRAGE syndrome by targeted Mendelian exome sequencing	2018	10.1186/s12881-018-0546-4	No keyword
Rajakumar V. *et al.*	Lymphoblastic lymphoma presenting as bilateral renal enlargement diagnosed by percutaneous kidney biopsy: Report of three cases	2016	10.4103/0971-4065.179368	No keyword
Robson H. *et al.*	Synthetic cannabis: adverse events reported to the New Zealand Pharmacovigilance Centre	2021	10.1080/15563650.2020.1828592	No keyword
Ferrè S. *et al.*	New insights into the role of HNF-1β in kidney (patho)physiology	2019	10.1007/s00467-018-3990-7	Review
Wente-Schulz S. *et al.*	Aetiology, course and treatment of acute tubulointerstitial nephritis in paediatric patients: a cross-sectional web-based survey	2021	10.1136/bmjopen-2020-047059	No keyword
Moradi A. *et al.*	The available clinical approaches to the management of patients with acute and chronic hypernatremia	2020	10.3889/oamjms.2020.4270	Review
Higham C.E. *et al.*	Hypopituitarism	2016	10.1016/S0140-6736(16)30053-8	Review
Sohail M.A. *et al.*	Ifosfamide-induced nephrogenic diabetes insipidus responsive to supraphysiologic doses of intravenous desmopressin	2021	10.5414/CNCS110589	No keyword
Jetton J.G. *et al.*	Pharmacological management of acute kidney injury and chronic kidney disease in neonates	2017	10.1016/j.siny.2016.09.002	Review
Bouchaala K. *et al.*	Acute Pancreatitis Induced by Diabetic Ketoacidosis with Major Hypertriglyceridemia: Report of Four Cases	2020	10.1155/2020/7653730	No keyword
Moor M.B. *et al.*	Ways of calcium reabsorption in the kidney	2016	10.1152/ajprenal.00273.2015	Review
van der Wijst J. *et al.*	Learning physiology from inherited kidney disorders	2019	10.1152/physrev.00008.2018	No keyword
Leonard N. *et al.*	Congenital nephrogenic diabetes insipidus in a preterm infant: Case presentation	2019	10.4183/aeb.2019.384	No keyword
Wang T. *et al.*	Arachidonic acid metabolism and kidney inflammation	2019	10.3390/ijms20153683	Review
Hyndman K.A. *et al.*	Fluid-electrolyte homeostasis requires histone deacetylase function	2020	10.1172/jci.insight.137792	No keyword

Iglesias P. *et al.*	Thyroid dysfunction and kidney disease: An update	2017	10.1007/s11154-016-9395-7	Review
Christ-Crain M. *et al.*	Diabetes insipidus	2019	10.1038/s41572-019-0103-2	No keyword
Nademi S. *et al.*	Protein misfolding in endoplasmic reticulum stress with applications to renal diseases	2019	10.1016/bs.apcsb.2019.08.001	No keyword
Resanović I. *et al.*	Hyperbaric Oxygen Therapy and Vascular Complications in Diabetes Mellitus	2020	10.1177/0003319720936925	Review
Brandoni A. *et al.*	Renal Expression and Urinary Excretion of Na-K-2Cl Cotransporter in Obstructive Nephropathy	2017	10.1155/2017/7171928	No keyword
Saleem S.F. *et al.*	Demographics	2019		No keyword
Umar U. *et al.*	Retrospective review of presentation of newly diagnosed children with diabetes mellitus in a Nigerian rural setting	2019	10.4103/mjdrdypu.mjdrdypu_227_18	Review
Chen A. *et al.*	Pediatric Renal Transplantation	2017	10.1016/B978-0-12-801734-0.00013-8	Book
Li Y. *et al.*	Prostaglandins in the pathogenesis of kidney diseases	2018	10.18632/oncotarget.25005	Review
Yatham L.N. *et al.*	Canadian Network for Mood and Anxiety Treatments (CANMAT) and International Society for Bipolar Disorders (ISBD) 2018 guidelines for the management of patients with bipolar disorder	2018	10.1111/bdi.12609	No keyword
Foreman J.W. *et al.*	Fanconi Syndrome	2019	10.1016/j.pcl.2018.09.002	Review
Skinner R. *et al.*	Late renal toxicity of treatment for childhood malignancy: risk factors	2018	10.1007/s00467-017-3662-z	Review
Shimazaki S. *et al.*	Acute kidney injury caused by ammonium acid urate crystals in diabetic ketoacidosis at the onset of type 1 diabetes mellitus	2021	10.1530/EDM-20-0143	No keyword
Westphal G.A. *et al.*	Brazilian guidelines for the management of brain-dead potential organ donors. The task force of the AMIB	2020	10.1186/s13613-020-00787-0	No keyword
Malakasioti G. *et al.*	Acute kidney injury in an infant with severe combined immunodeficiency: Questions	2019	10.1007/s00467-019-04302-x	Note
Roumelioti M.E. *et al.*	Principles of quantitative water and electrolyte replacement of losses from osmotic diuresis	2018	10.1007/s11255-018-1822-0	Review
Smith Z.R. *et al.*	Medication-Induced Hyperlactatemia and Lactic Acidosis: A Systematic Review of the Literature	2019	10.1002/phar.2316	Review
Bikbov B.T. *et al.*	Nephrology in Russia	2021	10.1007/978-3-030-56890-0_46	Book
Costa K.C.T. *et al.*	Systemic alterations induced by phospholipase A2	2018	10.1111/iep.12290	No keyword
Sas K.M. *et al.*	Hyperglycemia in the absence of cilia accelerates cystogenesis and induces renal damage	2015	10.1152/ajprenal.00652.2014	No keyword
Schönenberger D. *et al.*	Vhl deletion in renal epithelia causes HIF-1α-dependent	2016	10.18632/oncotarget.11275	No keyword
Jia Z. *et al.*	Role of COX-2/mPGES-1/prostaglandin E2 cascade in kidney injury	2015	10.1155/2015/147894	Review
Moritz M.L. *et al.*	Maintenance intravenous fluids in acutely Ill patients	2015	10.1056/NEJMra1412877	Review
Prozialeck W.C. *et al.*	Evaluation of cystatin C as an early biomarker of cadmium nephrotoxicity in the rat	2016	10.1007/s10534-015-9903-3	No keyword
Batlle D. *et al.*	Physiologic Principles in the Clinical Evaluation of Electrolyte	2013	10.1016/B978-0-12-381462-3.00074-4	Book
Brandt L.E. *et al.*	Localization of Canine	2012	10.1177/0300985811410720	Review
Kaplan N.M. *et al.*	Kaplan's clinical hypertension: Eleventh edition	2014		Book
Ashoor I.F. *et al.*	Physiology of the developing kidney: Fluid and electrolyte homeostasis and therapy of basic disorders (Na/H2O/K/Acid Base)	2015	10.1007/978-3-662-43596-0_12	Book
Burnstock G. *et al.*	Purinergic signalling in the kidney in health and disease	2014	10.1007/s11302-013-9400-5	Review
Obrig T.G. *et al.*	Shiga toxin pathogenesis: Kidney complications and renal failure	2012	10.1007/82_2011_172	Review
Patzer L. *et al.*	Nephrotoxicity as a cause of acute kidney injury in children	2008	10.1007/s00467-007-0721-x	Review
Skinner R. *et al.*	Nephrotoxicity of cancer treatment in children	2010	10.2217/phe.10.60	Review
Colvin R.B. *et al.*	Diagnostic Pathology: Kidney Diseases: A volume in Diagnostic Pathology	2015	10.1016/B978-0-323-37707-2.50001-X	Book
Prozialeck W.C. *et al.*	Kidney injury molecule-1 is an early biomarker of cadmium nephrotoxicity	2007	10.1038/sj.ki.5002467	No keyword
Ganguli A. *et al.*	Kidney diseases associated with haematological cancers	2015	10.1038/nrneph.2015.81	Review
Hogan J. *et al.*	Treatment of idiopathic FSGS with adrenocorticotropic hormone gel	2013	10.2215/CJN.02840313	No keyword
Riebeling C. *et al.*	Assaying embryotoxicity in the test tube: Current limitations of the embryonic stem cell test (EST) challenging its applicability domain	2012	10.3109/10408444.2012.674483	Review
Da Silva Junior G.B. *et al.*	New insights on pathophysiology	2011	10.1007/s00277-011-1327-8	Review
Chung E.Y. *et al.*	Nonsteroidal anti-inflammatory drug toxicity in children: A clinical review	2016	10.1097/PEC.0000000000000768	Review
Radi Z.A. *et al.*	Comparative Pathophysiology and Toxicology of Cyclooxygenases	2012	10.1002/9781118351918	Book
Conti G. *et al.*	Vitamin D intoxication in two brothers: Be careful with dietary supplements	2014	10.1515/jpem-2013-0220	No keyword
Snchez-Gonzlez P.D. *et al.*	An integrative view of the pathophysiological events leading to cisplatin nephrotoxicity	2011	10.3109/10408444.2011.602662	Review
Nasser H. *et al.*	Acute renal failure following Chlamydia pneumoniae pneumonia in a child	2010	10.1016/j.arcped.2010.05.003	No English
Januszewicz A. *et al.*	Malignant hypertension: New aspects of an old clinical entity	2016	10.20452/pamw.3275	Review
Taranta-Janusz K. *et al.*	Urine exoglycosidases are potential markers of renal tubular injury in children with ureteropelvic junction obstruction	2015	10.1111/apa.13068	No keyword

Niewczas M.A. *et al.*	Uremic solutes and risk of end-stage renal disease in type 2 diabetes: Metabolomic study	2014	10.1038/ki.2013.497	No keyword
Párraga A. *et al.*	Chemistry	2008	10.1002/9783527619337.ch10	Book
Kwon T.H. *et al.*	Renal aquaporins: Role in water balance disorders	2016		Book
Trachtman H. *et al.*	Renal and neurological involvement in typical Shiga toxin-associated HUS	2012	10.1038/nrneph.2012.196	Review
Lunøe M. *et al.*	Prevention of hospital-acquired hyponatraemia: Individualised fluid therapy	2015	10.1111/aas.12522	No keyword
Gebreselassie S *et al.*	Chemotherapy toxicities of the kidney	2011	10.1016/B978-1-4377-1015-1.00006-0	Book
Kutlu A.O. *et al.*	Rhabdomyolysis without detectable myoglobulinuria due to severe hypophosphatemia in diabetic ketoacidosis	2011	10.1097/PEC.0b013e31821dc68a	No keyword
Walker R.J. *et al.*	Cellular Mechanisms of Drug Nephrotoxicity	2013	10.1016/B978-0-12-381462-3.00085-9	Book
Kwon A.R. *et al.*	Hypodipsic hypernatremia leading to reversible renal failure following surgery for craniopharyngioma	2012	10.1515/jpem-2012-0176	No keyword
Moeller H.B. *et al.*	Renal aquaporins and water balance disorders	2016	10.1016/j.beem.2016.02.012	Review
Aronson J.K. *et al.*	Meyler's Side Effects of Drugs: The International Encyclopedia of Adverse Drug Reactions and Interactions	2015		Book
Kittanamongkolchai W. *et al.*	Efficacy and safety of adrenocorticotropic hormone treatment in glomerular diseases: A systematic review and meta-analysis	2016	10.1093/ckj/sfw045	Review
Pokorna P. *et al.*	The impact of hypothermia on the pharmacokinetics of drugs used in neonates and young infants	2015	10.2174/1381612821666150901110929	Review
Pesic S. *et al.*	Assessment of oxidative status in patients with acute kidney injury: A pilot study	2015	10.4077/CJP.2015.BAC243	No keyword
Han X. *et al.*	The role of taurine in renal disorders	2012	10.1007/s00726-012-1314-y	Review
O'Riordan E. *et al.*	Urine proteomics - Prospects for future diagnostics	2007	10.1556/APhysiol.94.2007.1-2.12	Review
Goligorsky M.S. *et al.*	Diagnostic potential of urine proteome: A broken mirror of renal diseases	2007	10.1681/ASN.2006121399	Review
Goebel J. *et al.*	Renal issues in organ transplant recipients in the PICU	2009	10.1007/978-3-540-74425-2_18	Book
van der Watt G. *et al.*	Laboratory investigation of the child with suspected renal disease	2015	10.1007/978-3-662-43596-0_19	Book
Chortis V. *et al.*	Miscellaneous Hormones	2015	10.1016/bs.seda.2015.07.004	Book
Constable P.D. *et al.*	Veterinary Medicine	2016	10.1016/B978-0-7020-5246-0.00027-9	Book
Li W. *et al.*	Two case reports of severe pediatric hyperosmolar hyperglycemia and diabetic ketoacidosis accompanied with rhabdomyolysis and acute renal failure	2014	10.1515/jpem-2014-0131	No keyword
Holt N.F. *et al.*	Vasopressin: A Review of Therapeutic Applications	2010	10.1053/j.jvca.2009.09.006	Review
Sharbaf F.G. *et al.*	Native nephrectomy prior to pediatric kidney transplantation: Biological and clinical aspects	2012	10.1007/s00467-012-2115-y	No keyword
Balestracci A. *et al.*	Ibuprofen-associated acute kidney injury in dehydrated children with acute gastroenteritis	2015	10.1007/s00467-015-3105-7	No keyword
Niscola P. *et al.*	Management of hematological malignancies in patients affected by renal failure	2011	10.1586/era.11.2	Review
Kemter E. *et al.*	Type of uromodulin mutation and allelic status influence onset and severity of uromodulinassociated kidney disease in mice	2013	10.1093/hmg/ddt263	No keyword
Nanau R.M. *et al.*	Ibuprofen: Associated adverse drug reactions	2013		Book
Bockenhauer D. *et al.*	The kidney speaks: Interpreting urinary sodium and osmolality	2011	10.1136/archdischild-2011-300115	Review
ZhouXin J. *et al.*	Renal changes with aging and end-stage renal disease	2014		Book
Ruskoaho H. *et al.*	Atrial natriuretic peptide: Synthesis	1992		Review
Abramowicz D. *et al.*	Does pre-emptive transplantation versus post start of dialysis transplantation with a kidney from a living donor improve outcomes after transplantation? A systematic literature review and position statement by the Descartes Working Group and ERBP	2016	10.1093/ndt/gfv378	Review
Ulinski T. *et al.*	Acute tubulointerstitial nephritis	2012	10.1007/s00467-011-1915-9	Review
Ellis D. *et al.*	Regulation of Fluids and Electrolytes	2016	10.1016/B978-0-323-34125-7.00005-X	Book
Sun Y. *et al.*	Inhibition of mitochondrial complex-1 restores the downregulation of aquaporins in obstructive nephropathy	2016	10.1152/ajprenal.00215.2015	No keyword
Zieg J. *et al.*	Oral penicillin-associated acute kidney injury in an infant with acute pyelonephritis	2015	10.1111/ped.12571	No keyword
Perazella M.A. *et al.*	Nephrotoxicity from chemotherapeutic agents: Clinical manifestations	2010	10.1016/j.semnephrol.2010.09.005	Review
Basavaraj B. *et al.*	A study on the clinic-epidemiological profile of patients presenting to the emergency department with acute kidney injury in a tertiary care hospital	2024	10.48047/jcdr.2024.15.06.81	No keyword
Masood A. *et al.*	Cystinosis Presenting with Complications in an Adult: A Difficult Case	2024		Conference Abstract
Pieri G.R. *et al.*	Tubulointerstitial acute kidney injury in paediatrics: a single center experience	2023	10.1007/s00467-023-06094-7	Conference Abstract
Bell E. *et al.*	Atypical Presentation of New Onset Diabetes with Hyperglycemic Hyperosmolar State in a Toddler	2023	10.1159/000531602	Conference Abstract
Egodawaththe N.S. *et al.*	NSAID-induced acute tubular interstitial nephritis in children: do we need more regulation?	2023	10.1136/archdischild-2023-rcpch.129	Conference Abstract
Ahlenstiel-Grunow T. *et al.*	Lifelong Polyuria Continues Post-Kidney Transplant in Teenagers with End Stage Kidney Disease; Lessons Learned and Need for Newer Strategies Beyond Native Nephrectomies	2023	10.1016/j.ajt.2023.05.014	Conference Abstract

Evers K. *et al.*	Acute kidney injury in diabetic ketoacidosis in children and adolescents	2022		Conference Abstract
Andrade A. *et al.*	Liposomal amphotericin b nephrotoxicity in children: a cross-sectional analysis	2022	10.1007/s00467-022-05630-1	Conference Abstract
Turtsevich I. *et al.*	Role of the paediatric rheumatologist in the management of tubulointerstitial nephritis and uveitis	2022	10.1186/s12969-022-00729-z	Conference Abstract
Hayashi A. *et al.*	Selective Intrauterine Growth Restriction in a Monochorionic Diamniotic Twin Having Placental Features Similar to Monochorionic Monoamniotic Twins: A Case Report	2021	10.1016/j.placenta.2021.08.025	Conference Abstract
Long N. *et al.*	Approaching High-Metabolic Risk Youth During a Pandemic: Severe Presentations of New Onset Type 2 Diabetes	2021	10.1210/jendso/bvab048.1418	Conference Abstract
Alloah Q. *et al.*	An unusual presentation of t-cell acute lymphoblastic leukemia: Syncope and hypercalcemia of malignancy	2021	10.1542/peds.147.3_MeetingAbstract.850	Conference Abstract
Bouchama F.S. *et al.*	Hyponatremic hypertensive syndrome (hhs) in an 8-monthold child presenting as status epilepticus and hypertensive crisis in pediatric intensive care unit (picu): A case report	2021	10.1097/01.pcc.0000741252.18521.82	Conference Abstract
Shen C.L. *et al.*	Renal replacement therapy in an adolescent with hyperosmolar hyperglycemic state	2021	10.1159/000515882	Conference Abstract
Adapa G. *et al.*	A case of central diabetes insipidus as a cause of unexplained hydroureteronephrosis	2019		Conference Abstract
Baumann H. *et al.*	Altered mental status presenting to emergency department	2019	10.1136/jim-2018-000974.318	Conference Abstract
Umar U. *et al.*	Pattern of presentation of newly diagnosed children with diabetes mellitus in a rural setting	2018	10.1111/pedi.12746	Conference Abstract
Parmar R.S. *et al.*	Cerebral infarction in paediatric diabetic ketoacidosis	2017	10.1136/archdischild-2017-313087.158	Conference Abstract
Ariyawatkul K. *et al.*	DKA with severe acute kidney injury in an adolescent boy: A case report	2017	10.1186/s13633-017-0054-x	Conference Abstract
Verma A. *et al.*	Incidence and Characteristics of Acute Kidney Injury in Children and Adolescents with Diabetic Ketoacidosis	2016		Conference Abstract
Bukkar F. *et al.*	Acute kidney injury as a severe complication of diabetic ketoacidosis in children: A case report	2013		Conference Abstract
Stavileci V.N. *et al.*	Acute tubulointestitial nephritis caused by ascaridosis-first case recovered from dialyses in Kosova	2013	10.1007/s00467-013-2518-4	Conference Abstract
Hubscher C.H. *et al.*	Impact of activity-based recovery training and desmopressin on spinal cord injury-induced polyuria in Wistar rats	2023	10.1080/10790268.2022.2069538.	No keyword
Siegel N.J. *et al.*	Disorders of urine volume in the critically ill child	1984	Yale J Biol Med	Review
Perfumo F. *et al.*	[Nephronophthisis]	1976	Minerva Nefrol	Review
Jones D.P. *et al.*	Renal toxicity of cancer chemotherapeutic agents in children: ifosfamide and cisplatin	1995	10.1097/00008480-199504000-00016.	No keyword
Galaske R.G. *et al.*	[Primary polyuric kidney failure and acute yellow liver dystrophy following infusion of glucose substitutes in children]	1986	10.1055/s-2008-1068568.	No keyword
Adeleke S.I. *et al.*	Childhood diabetes mellitus in Kano, North West, Nigeria	2010	10.4314/njm.v19i2.56502.	No keyword
Onay O.S. *et al.*	Acute, reversible nonoliguric renal failure in two children associated with analgesic-antipyretic drugs	2009	10.1097/PEC.0b013e31819e38d4.	No keyword
Stone H.H. *et al.*	Renal decapsulation in the prevention of post-ischemic oliguria	1977	10.1097/00000658-197709000-00012.	No keyword
Mandal A.K. *et al.*	Diagnosis and management of acute renal failure (acute uraemia)	1976	J Indian Med Assoc	No keyword
Gordon M. *et al.*	Acute renal papillary necrosis with complete bilateral ureteral obstruction in a child	2007	10.1016/j.urology.2007.01.022.	No keyword
Carrillo-Esper R. *et al.*	[Rhabdomyolysis and acute renal failure in human influenza A H1N1 mediated infection]	2009	Gac Med Mex	No keyword
Straub E. *et al.*	[Thyroxine treatment in acute renal failure (author's transl)]	1975		No keyword
Callis L. *et al.*	[Renal hypoplasia with oligonephronia]	1970		No keyword
O'Regan S. *et al.*	Hemolytic uremic syndrome: urate nephropathy superimposed on an acute glomerulopathy? An hypothesis	1988		No keyword
Markiewicz A. *et al.*	Urine antitrypsin activity (UAA) in the course of acute renal failure	1971		No keyword
Markiewicz A. *et al.*	[Urine antitrypsin activity (UAA) in acute renal failure]	1970		No keyword
Brunner L. *et al.*	[Causes of acute kidney failure following surgery using the heart-lung machine. (Report on 43 out of 1500 case histories of patients operated during extracorporeal circulation)]	1972	10.1055/s-0028-1098971.	No keyword
Markiewicz A. *et al.*	[Urokinase (UK) activity in acute renal failure]	1970		No keyword
Grabensee B. *et al.*	[A 28-eight-year-old woman manifests immediately after the birth of her first child oligoanuria for 2 days, then polyuria for the next 6 days with increased creatinine to max. 5.1 mg/dl, to the 7th puerperal day with complete remission]	1993		No keyword
